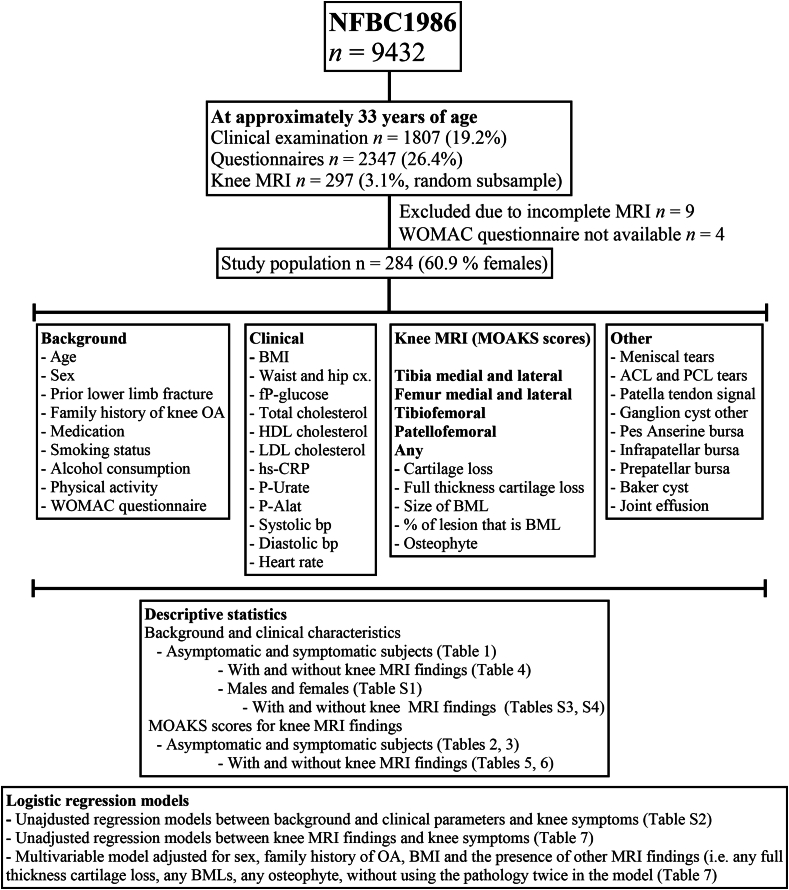# Associations of clinical measures and structural knee magnetic resonance imaging findings with knee symptoms in a birth cohort of 33-year-old adults

**DOI:** 10.1016/j.ocarto.2025.100730

**Published:** 2025-12-13

**Authors:** Antti Kemppainen, Joona Tapio, Miika T. Nieminen, Simo Saarakkala, Mika T. Nevalainen

**Affiliations:** a Research Unit of Health Sciences and Technology, Faculty of Medicine, University of Oulu, P.O. Box 5000, FI-90014 Oulu, Finland; b Department of Diagnostic Radiology, Oulu University Hospital, P.O. Box 50, FI-90029 Oulu, Finland; c Faculty of Biochemistry and Molecular Medicine, University of Oulu, P.O. Box 5400, FIN-90014 Oulu, Finland; d Biocenter Oulu, University of Oulu, P.O. Box 5400, FIN-90014 Oulu, Finland; e Medical Research Center Oulu, University of Oulu and Oulu University Hospital, Oulu, Finland

**Keywords:** Cohort studies, Knee joint /diagnostic imaging, Magnetic resonance imaging, Osteoarthritis, Knee /diagnostic imaging

## Abstract

**Objective:**

To investigate the associations between knee magnetic resonance imaging (MRI) findings, knee joint symptoms and related health factors in a random subpopulation of 33-year-olds from the Northern Finland 1986 Birth Cohort.

**Method:**

Subjects with questionnaire, knee MRI, and clinical symptom data, were included in this study (n = 284, 60.9 % females, age 33.7 years). Knee MRI data was graded using the MRI Osteoarthritis Knee Score (MOAKS) system. Symptoms were evaluated using the Visual Analogue Scale (VAS) version of the Western Ontario and McMaster Universities Osteoarthritis Index (WOMAC), and participants with VAS >3 in any WOMAC sub-question were considered symptomatic.

**Results:**

12.2 % (74.3 % females) of the study population were symptomatic, and were more likely to have a higher BMI, adverse lipid profile and a family history of knee OA. Advanced MRI findings were sparse. Most MRI findings, such as bone marrow lesions (BMLs) and osteophytes, were more prevalent and severe in symptomatic participants. In adjusted regression analyses, BMLs in the medial femoral compartment and osteophytes in the lateral tibial and medial femoral compartments, patellar tendon signal and joint effusion were associated with knee symptoms.

**Conclusion:**

Several MRI findings, most notably medial femoral BMLs, osteophytes, patellar tendinopathy and joint effusion were associated with knee symptoms. Of other factors, sex, family history and clinical factors such as body composition are associated with knee joint symptomatology from early adulthood.

## 1 Introduction

Osteoarthritis (OA) is the most prevalent form of arthritis in the adult population [1]. The knee joint is most frequently affected, with the most typical symptoms being persistent knee pain and a slow decline in joint mobility [1,2]. Pain is often worse when, for example, using stairs, and joint stiffness is worse in the morning and after prolonged inertness. Based on patient history and physical examination, a presumptive diagnosis of knee OA can be made [2]. Conventional radiographs are obtained when necessary, and either clear radiographic OA or a lack of alternative diagnoses is expected [[2], [3], [4]]. Radiographs alone are often discordant with patient-reported symptoms [5], and do not exclude knee OA [2].

In terms of structural visualization of joint tissues, magnetic resonance imaging (MRI) is superior to radiographs. MRI enables the direct visualization of, for example, cartilage lesions, the menisci and bone marrow lesions (BMLs), and is the gold standard imaging modality for research purposes [3,6]. Although seldom indicated [2], knee MRI is increasingly utilized in patient workup in part due to reduced knee arthroscopy rates, but also unwarrantedly [7,8]. This increase could partly explain the observed and the projected increase of global knee OA incidence [1,9,10].

Previous studies have reported that MRI findings consistent with knee OA are common in both symptomatic and asymptomatic subjects [11,12]. Knee pain has been associated with several MRI findings such as synovitis [[13], [14], [15]], effusion [13,15,16], BMLs [15,17], meniscal pathology [13,16,18], osteophytes [18] and full-thickness [18] as well as progressive cartilage lesions [16]. However, these studies have been mainly conducted in middle-aged and elderly subjects in whom definite knee OA is already prevalent [1]. Only few studies in younger populations without obvious OA risk factors have been published [[19], [20], [21]], and small osteophytes are also common in younger populations [[21], [22], [23]].

In our recent study, we described the knee MRI findings and their associated background and clinical parameters in a random subpopulation of 33-year-olds of the Northern Finland 1986 Birth Cohort (NFBC1986) [21]. OA-associated findings, such as small cartilage lesions and osteophytes, were frequent in this relatively young and healthy study population, with some knee joints already having definite knee OA [24,25], but most participants being asymptomatic [21]. Higher body mass index (BMI) and a family history of OA were associated with the most imaging findings [21]. The present study is a continuation of our recent study and focuses on the reported joint symptoms by evaluating their associations with imaging and related lifestyle and health factors.

## 2 Method

### 2.1 Study population

The NFBC1986 is a longitudinal general population-based birth cohort consisting of 99 % of children who were due to be born in the two northernmost provinces of Finland between July 1985 and June 1986 (*n* = 9432). The latest data collection was at the age of 33 years [26]. Knee MRI was conducted for a random subsample (*n* = 297) with no other selection criteria. Nine MRIs were incomplete and subsequently excluded. The knee with more symptoms or the dominant knee in lack of symptoms [77.1 % (*n* = 222) right knee, 22.9 % (*n* = 66) left knee] was imaged [27]. Standard anteroposterior, lateral and mountain view radiographs of the same knee joint were also imaged for the whole subpopulation by authorized research personnel. For this study, the final study population consisted of subjects with available clinical and postal questionnaire data (*n* = 288), complete knee MRI (*n* = 288) and radiograph (*n* = 288) data and data on clinical symptoms of the knee (*n* = 284) at 33 years of age. A flow-chart of the study population is presented in Fig. S1.

### 2.2 Background and clinical characteristics

The methods for laboratory and clinical measures used have been recently described in detail [21]. Background information was determined by postal questionnaires. Subjects self-reported their medical history, prior lower limb fractures, family history of knee OA (parents, siblings and grandparents, classed as “no” or “yes”), medications, smoking status (“never” or “ever”), and the frequency and number of alcoholic beverages consumed. “Physical activity score” was based on four questions: How long do you perform light and heavy exercise at a time (none (0) to over 1.5 h (6)) and how often (once a month at most (0) to daily (6)), respectively, and the sum was used in the analyses as a continuous variable.

Trained nurses measured body height and weight, waist and hip circumference, brachial systolic and diastolic blood pressure (Sbp and Dbp) and heart rate. Body mass index (BMI) was calculated. Laboratory analyses were conducted from fresh blood samples after overnight fasting.

The Visual Analogue (VAS) version of The Western Ontario and McMaster Universities Osteoarthritis Index (WOMAC) [28,29] scale was filled prior to knee MRI [21]. Participants marked each answer on a 100-mm VAS scale, which were subsequently measured documented and scored from 0 to 10. Subscale scores range from 0 to 50 (pain), 0 to 20 (stiffness) and 0 to 170 (physical function). The pain category included five sub-questions on pain during walking, using stairs, in bed, sitting/lying down, and standing. The stiffness category had two sub-questions on morning and daytime knee joint stiffness. The physical function category comprised 17 sub-questions on difficulties when descending stairs, ascending stairs, standing up, standing, bending, walking, getting in and out of car, shopping, putting on socks, getting out of bed, taking off socks, lying in bed, getting in and out of bath, sitting, getting on and off the toilet, doing heavy chores and doing light chores.

### 2.3 Imaging

Knee MRI and the semi-quantitative scoring of MRIs were done as previously described in detail [21]. For each participant, one knee joint was imaged using 3T MRI with identical imaging protocols and scored using the MRI Osteoarthritis Knee Score (MOAKS) system [30,31]. A musculoskeletal radiology fellow with five years of training (AK) did the scoring after calibration sessions with a board-certified fellowship-trained musculoskeletal radiologist with ten years of experience (MTNe). From the radiographs, tibiofemoral OA was evaluated (AK) using the Kellgren-Lawrence (KL) classification [32], with medial and lateral KL grade being recorded separately for each knee joint.

### 2.4 Statistical methods

Subjects with missing data were omitted from individual analyses. Prior to variance analysis, continuous variables were checked for skewness with histograms. Normally distributed continuous variables are presented as mean (M) and standard deviation (SD), while non-normally distributed continuous variables are presented as median (Mn) and interquartile range. Count data is presented as number of observations (*n*) and percentage.

To approximate the prevalence of definite OA with Hunter's classification [24,25], we defined tibiofemoral OA as: Any tibiofemoral osteophyte MOAKS ≥2 + any tibiofemoral full-thickness cartilage loss, or either of the previous and two of the following: Any BML, horizontal meniscal tear or maceration, any cartilage loss MOAKS ≥2. Patellofemoral OA was defined as patellofemoral osteophyte MOAKS ≥2 + either patellofemoral cartilage loss MOAKS ≥2 or full-thickness cartilage loss.

Background and MRI data were first evaluated with descriptive statistics in groups determined by WOMAC scores. Participants with VAS >3 in any sub-question were considered “symptomatic” whereas participants with VAS <3 in all sub-questions were considered “asymptomatic”, similarly to Ref. [33].

The odds ratios of clinical and background variables and MRI findings on symptoms were first evaluated with unadjusted logistic regression models (categorized outcome variable: “symptomatic” or “asymptomatic”). Some MRI parameters (especially advanced findings such as BMLs and full-thickness cartilage lesions) included <5 cases, possibly giving unreliable risk estimates with wide confidence intervals (CIs). As advanced MRI findings have been previously associated with clinical symptoms, these risk estimates were reported regardless. For colinear explanatory variables (such as Sbp and Dbp) only one of the parameters was analyzed.

Subjects were then divided into four subclasses (−/−, +/−, +/+, −/−) based on knee symptoms (- or +) and MRI findings (+ or -). MRI findings included cartilage loss (grade >1), osteophyte (grade >1), any BML, any full-thickness cartilage loss, any meniscal tear or maceration, popliteal cyst, tears and repairs of anterior (ACL) and posterior cruciate ligament and joint effusion (grade >1). Clinical and MRI data were then evaluated in descriptive statistics according to these classes.

As an adjusted model, the odds ratios of each knee MRI finding on symptoms were adjusted for sex, family history of OA, BMI and the presence of other MRI findings (*i.e*. any full-thickness cartilage loss, any BMLs, any osteophyte, without using the given structural pathology twice in the model). The adjustments based on the descriptive data and results from the unadjusted regression analyses.

Statistical analyses were performed using IBM SPSS statistics, version 29.0.0.0 (IBM Corp, Armonk, NY).

## 3 Results

### 3.1 Background and clinical characteristics of symptomatic and asymptomatic subjects

Almost 75 % of symptomatic subjects were female, compared to 59 % of asymptomatic subjects (Table 1). Mean WOMAC scores were higher in symptomatic subjects, being still relatively low with a mean total score of 36/240 (VAS scale, Table 1). Of all symptomatic participants, 57 % reported VAS >3 on pain when using stairs and 26 % and 29 % reported morning and daytime joint stiffness, respectively, with the percentage of reported symptoms in other sub-questions ranging from 3 % to 20 %, as shown in Ref. [21]. Symptomatic males had slightly more severe symptoms than females (Table S1). Family history was more common in symptomatic subjects (54.3 %) compared to asymptomatic (30.1 %) and was more common for females compared to males (Table S1). BMI and waist/hip circumferences were higher in the symptomatic subjects. No major differences were observed in laboratory or clinical measures, while slightly higher triglycerides, hs-CRP and heart rate were observed in the symptomatic subjects (Table 1).

**Table 1:** Background characteristics in symptomatic and asymptomatic subjects.

	Asymptomatic	Symptomatic
Number of participants n (%)	249 (100.0)	35 (100.0)
Males n (%)	102 (41.0)	9 (25.7)
Females n (%)	147 (59.0)	26 (74.3)
Age (years)	33.7 (0.4)	33.7 (0.4)
WOMAC pain	1.4 (1.9)	10.2 (7.0)
WOMAC stiffness	0.6 (1.0)	4.4 (4.0)
WOMAC function	2.5 (4.4)	22 (21.6)
WOMAC total	4.5 (6.5)	36.2 (29.3)
Prior lower limb fracture n (%)	35 (14.1)	2 (5.7)
Family history of knee OA n (%)	75 (30.1)	19 (54.3)
Anti-inflammatory medication n (%)	6 (2.4)	0 (0.0)
Strong pain medication (%)	14 (5.6)	3 (8.6)
Ever smoker n (%)	153 (61.4)	23 (65.7)
Alcohol consumption g/week	16.5 (10.5–45.0)	16.5 (12.0–31.5)
Physical activity score	14.9 (3.3)	14.4 (3.8)
BMI (kg/mˆ2)	25.5 (4.5)	26.7 (5.1)
Waist circumference (cm)	86.4 (13.1)	89.9 (13.6)
Hip circumference (cm)	99.4 (11.6)	101.4 (11.2)
fP-Glucose (mmol/L)	5.0 (4.7–5.2)	4.9 (4.8–5.2)
fP-total cholesterol (mmol/L)	4.7 (0.9)	4.6 (0.9)
fP-HDL cholesterol (mmol/L)	1.5 (0.3)	1.5 (0.4)
fP-LDL cholesterol (mmol/L)	2.7 (0.8)	2.8 (0.9)
fP-triglycerides (mmol/L)	0.9 (0.7)	1.1 (0.5)
hs-CRP (mg/l)	0.7 (0.4–1.6)	1.1 (0.5–2.3)
P-Urate (umol/L)	305.9 (70.4)	297.6 (72.1)
Systolic blood pressure (mmHg)	112.1 (12.2)	111.2 (12.3)
Diastolic blood pressure (mmHg)	74.1 (8.8)	75.6 (9.4)
Heart rate (bpm)	71.9 (12.4)	73.7 (12.4)

Asymptomatic = VAS <3 in all WOMAC sub-questions, symptomatic = VAS >3 in any WOMAC sub-question.

Subsequently, we evaluated unadjusted associations of individual clinical parameters with knee symptoms in logistic regression models (Table S2). In whole population analysis, although female sex and a family history seemed to increase the risk for symptoms, no significant associations were observed (Table S2). In males, HDL cholesterol was negatively associated with symptoms (Table S2). In females, having a family history and higher triglyceride levels were associated with symptoms, while a trend was also observed for greater waist circumference (Table S2).

Radiographic OA findings were rare in the study population, with KL grade 0 in 83 %, grade 1 in 14 %, grade 2 in 2 % and grade 3 in 1 % of the participants (Table S3).

### 3.2 Most knee MRI findings are more common in symptomatic subjects than asymptomatic subjects

Prevalence and severity of the most severe cartilage lesions, BMLs and osteophytes in symptomatic and asymptomatic subjects are presented in Table 2. In the tibiofemoral joint, the percentages of joint quadrants with no cartilage lesions ranged between 81.9 and 96.5 % in the asymptomatic and 75.3–91.4 % in the symptomatic. In total, tibiofemoral cartilage lesions were found in 24.5 % of asymptomatic and 34.3 % of symptomatic joints. Most tibiofemoral cartilage lesions were small. Higher grade lesions, full-thickness lesions and BMLs were rare but more common in symptomatic joints. Most tibiofemoral osteophytes were small or doubtful. Higher grade tibiofemoral osteophytes were more common in symptomatic subjects (Table 2).

**Table 2:** Counts of the most severe in-patient MRI-detected cartilage lesions, BMLs and osteophytes in the tibiofemoral and patellofemoral joint regions in symptomatic and asymptomatic subjects.

Site	Tibia medial		Tibia lateral		Femur medial		Femur lateral		Tibiofemoral		Patellofemoral		Any	
**Symptoms**	No	Yes	No	Yes	No	Yes	No	Yes	No	Yes	No	Yes	No	Yes
**Cartilage loss**														
0 (none)	240 (96.4)	31 (88.6)	230 (92.4)	27 (77.1)	204 (81.9)	26 (74.3)	235 (94.4)	32 (91.4)	188 (75.5)	23 (65.7)	111 (44.6)	12 (34.3)	91 (36.5)	10 (28.6)
1 (<10 %)	7 (2.8)	4 (11.4)	17 (6.8)	6 (17.1)	31 (12.4)	3 (8.6)	10 (4.0)	2 (5.7)	43 (17.3)	6 (17.1)	91 (36.5)	13 (37.1)	98 (39.4)	14 (40)
2 (<10–75 %)	2 (0.8)	0 (0.0)	2 (0.8)	2 (5.7)	14 (5.6)	5 (14.3)	4 (1.6)	0 (0.0)	18 (7.2)	4 (11.4)	40 (16.1)	9 (25.7)	53 (21.3)	9 (25.7)
3 (>75 %)	0 (0.0)	0 (0.0)	0 (0.0)	0 (0.0)	0 (0.0)	1 (2.9)	0 (0.0)	1 (2.9)	0 (0.0)	2 (5.7)	7 (2.8)	1 (2.9)	7 (2.8)	2 (5.7)
**Full thick. cartilage loss**														
0 (none)	248 (99.6)	34 (97.1)	245 (98.4)	33 (94.3)	244 (98.0)	31 (88.6)	243 (97.6)	34 (97.1)	236 (94.8)	30 (85.7)	222 (89.2)	29 (82.9)	211 (84.7)	27 (77.1)
1 (<10 %)	0 (0.0)	1 (2.9)	4 (1.6)	2 (5.7)	2 (0.8)	2 (5.7)	4 (1.6)	0 (0.0)	9 (3.6)	2 (5.7)	23 (9.2)	3 (8.6)	30 (12.0)	3 (8.6)
2 (<10–75 %)	1 (0.4)	0 (0.0)	0 (0.0)	0 (0.0)	2 (0.8)	2 (5.7)	2 (0.8)	1 (2.9)	3 (1.2)	3 (8.6)	4 (1.6)	2 (5.7)	7 (2.8)	4 (11.4)
3 (>75 %)	0 (0.0)	0 (0.0)	0 (0.0)	0 (0.0)	1 (0.4)	0 (0.0)	0 (0.0)	0 (0.0)	1 (0.4)	0 (0.0)	0 (0.0)	1 (2.9)	1 (0.4)	1 (2.9)
**Size of BML N(%)**														
0 (none)	248 (99.6)	34 (97.1)	245 (98.4)	33 (94.3)	246 (98.8)	31 (88.6)	245 (98.4)	34 (97.1)	238 (95.6)	30 (85.7)	229 (92.0)	30 (85.7)	218 (87.6)	27 (77.1)
1 (<33 %)	0 (0.0)	1 (2.9)	4 (1.6)	2 (5.7)	3 (1.2)	2 (5.7)	3 (1.2)	1 (2.9)	9 (3.6)	3 (8.6)	17 (6.8)	3 (8.6)	26 (10.4)	5 (14.3)
2 (33–66 %)	1 (0.4)	0 (0.0)	0 (0.0)	0 (0.0)	0 (0.0)	1 (2.9)	1 (0.4)	0 (0.0)	2 (0.8)	1 (2.9)	2 (0.8)	2 (5.7)	4 (1.6)	2 (5.7)
3 (>66 %)	0 (0.0)	0 (0.0)	0 (0.0)	0 (0.0)	0 (0.0)	1 (2.9)	0 (0.0)	0 (0.0)	0 (0.0)	1 (2.9)	1 (0.4)	0 (0.0)	1 (0.4)	1 (2.9)
**% of lesion that is BML**														
0 (none)	248 (99.6)	34 (97.1)	245 (98.4)	33 (94.3)	246 (98.8)	31 (88.6)	245 (98.4)	34 (97.1)	238 (95.6)	30 (85.7)	232 (93.2)	30 (85.7)	221 (88.8)	27 (77.1)
1 (<33 %)	0 (0.0)	0 (0.0)	0 (0.0)	0 (0.0)	0 (0.0)	0 (0.0)	0 (0.0)	0 (0.0)	0 (0.0)	0 (0.0)	0 (0.0)	0 (0.0)	0 (0.0)	0 (0.0)
2 (33–66 %)	0 (0.0)	0 (0.0)	0 (0.0)	0 (0.0)	0 (0.0)	1 (2.9)	0 (0.0)	0 (0.0)	0 (0.0)	1 (2.9)	1 (0.4)	0 (0.0)	1 (0.4)	0 (0.0)
3 (>66 %)	1 (0.4)	1 (2.9)	4 (1.6)	2 (5.7)	3 (1.2)	3 (8.6)	4 (1.6)	1 (2.9)	11 (4.4)	4 (11.4)	16 (6.4)	5 (14.3)	27 (10.8)	8 (22.9)
**Osteophyte**														
0 (none)	235 (94.4)	30 (85.7)	233 (93.6)	29 (82.9)	238 (95.6)	28 (80.0)	217 (87.1)	29 (82.9)	207 (83.1)	27 (77.1)	124 (49.8)	14 (40.0)	114 (45.8)	14 (40.0)
1 (small)	12 (4.8)	2 (5.7)	15 (6.0)	2 (5.7)	9 (3.6)	4 (11.4)	29 (11.6)	3 (8.6)	37 (14.9)	4 (11.4)	112 (45.0)	15 (42.9)	121 (48.6)	14 (40.0)
2 (medium)	1 (0.4)	2 (5.7)	1 (0.4)	3 (8.6)	1 (0.4)	2 (5.7)	2 (0.8)	2 (5.7)	4 (1.6)	3 (8.6)	12 (4.8)	5 (14.3)	13 (5.2)	6 (17.1)
3 (large)	1 (0.4)	1 (2.9)	0 (0.0)	1 (2.9)	1 (0.4)	1 (2.9)	1 (0.4)	1 (2.9)	1 (0.4)	1 (2.9)	1 (0.4)	1 (2.9)	1 (0.4)	1 (2.9)

Asymptomatic = VAS <3 in all WOMAC sub-questions, symptomatic = VAS >3 in any WOMAC sub-question.

In the patellofemoral joint, cartilage lesions were found in 55.4 % of asymptomatic joints and 65.7 % of symptomatic joints, and more severe lesions were more common in symptomatic subjects. No clear difference in patellofemoral full-thickness cartilage lesions or BMLs were observed between asymptomatic and symptomatic subjects. Patellofemoral osteophytes were common, with the more severe findings being more common in symptomatic subjects compared to asymptomatic subjects (Table 2).

Other MRI features evaluated are presented in Table 3. Meniscal findings were more common in symptomatic subjects, mostly accounted for by intrameniscal signal. Meniscal tears and macerations were rare in both groups. Increased patellar tendon signal was identified in 14.3 % of symptomatic subjects compared to 2.8 % in the asymptomatic. Increased infrapatellar signal in T2-weighted images was seen in 22.9 % of symptomatic and 16.5 % of asymptomatic subjects. Popliteal cysts were more common in symptomatic subjects (54.3 %) compared to asymptomatic (36.5 %), and severe knee effusion was more frequent in symptomatic subjects (Table 3).

**Table 3:** Meniscal morphology, other parameters of interest and prevalence and severity of knee joint effusion in symptomatic and asymptomatic subjects.

Medial meniscal morphology	Anterior		Body		Posterior			
Symptoms	No	Yes	No	Yes	No	Yes		
Normal	244 (98.0)	33 (94.3)	218 (87.6)	28 (80.0)	220 (88.4)	27 (77.1)		
Intrameniscal signal	2 (0.8)	1 (2.9)	17 (6.8)	4 (11.4)	17 (6.8)	5 (14.3)		
Vertical tear	0 (0.0)	0 (0.0)	0 (0.0)	0 (0.0)	0 (0.0)	0 (0.0)		
Horizontal tear	1 (0.4)	0 (0.0)	10 (4.0)	1 (2.9)	10 (4.0)	1 (2.9)		
Radial tear	0 (0.0)	0 (0.0)	0 (0.0)	0 (0.0)	0 (0.0)	0 (0.0)		
Complex tear	2 (0.8)	0 (0.0)	3 (1.2)	1 (2.9)	1 (0.4)	1 (2.9)		
Partial maceration	0 (0.0)	1 (2.9)	1 (0.4)	1 (2.9)	1 (0.4)	1 (2.9)		
Total maceration	0 (0.0)	0 (0.0)	0 (0.0)	0 (0.0)	0 (0.0)	0 (0.0)		

Lateral meniscal morphology	**Anterior**		**Body**		**Posterior**			

Symptoms	No	Yes	No	Yes	No	Yes		

Normal	245 (98.4)	35 (100.0)	247 (99.2)	34 (97.1)	245 (98.4)	34 (97.1)		
Intrameniscal signal	1 (0.4)	0 (0.0)	0 (0.0)	0 (0.0)	3 (1.2)	0 (0.0)		
Vertical tear	0 (0.0)	0 (0.0)	0 (0.0)	0 (0.0)	0 (0.0)	0 (0.0)		
Horizontal tear	2 (0.8)	0 (0.0)	1 (0.4)	0 (0.0)	1 (0.4)	1 (2.9)		
Radial tear	0 (0.0)	0 (0.0)	0 (0.0)	0 (0.0)	0 (0.0)	0 (0.0)		
Complex tear	1 (0.4)	0 (0.0)	1 (0.4)	1 (2.9)	0 (0.0)	0 (0.0)		
Partial maceration	0 (0.0)	0 (0.0)	0 (0.0)	0 (0.0)	0 (0.0)	0 (0.0)		
Total maceration	0 (0.0)	0 (0.0)	0 (0.0)	0 (0.0)	0 (0.0)	0 (0.0)		

**Other parameters of interest**	**Present**	**Present**						

Symptoms	No	Yes						

ACL tear	2 (0.8)	0 (0.0)						
ACL repair	0 (0.0)	2 (5.7)						
PCL tear	2 (0.8)	1 (2.9)						
PCL repair	0 (0.0)	0 (0.0)						
Patellar tendon signal	7 (2.8)	5 (14.3)						
Any ganglion cyst	56 (22.5)	7 (20.0)						
Pes anserine bursitis	2 (0.8)	0 (0.0)						
Infrapatellar bursa signal	41 (16.5)	8 (22.9)						
Prepatellar bursa signal	84 (33.7)	11 (31.4)						
Popliteal cyst	91 (36.5)	19 (54.3)						

**OA based on Hunter's definition**	**Present**	**Present**						

Symptoms	No	Yes						

TF joint	3 (1.2)	4 (11.4)						
PF joint	5 (2.0)	5 (14.3)						

	**None**		**Small**		**Medium**		**Large**	

Symptoms	No	Yes	No	Yes	No	Yes	No	Yes

Joint effusion	148 (59.4)	14 (40.0)	83 (33.3)	14 (42.9)	17 (6.8)	4 (11.4)	1 (0.4)	2 (5.7)

Asymptomatic = VAS <3 in all WOMAC sub-questions, symptomatic = VAS >3 in any WOMAC sub-question. ACL; Anterior Cruciate Ligament, PCL; Posterior Cruciate Ligament.

Concerning definite knee OA [24], a total of seven subjects had tibiofemoral OA and ten had patellofemoral OA. In asymptomatic subjects, three (1.2 %) had tibiofemoral and five (2.0 %) had patellofemoral OA. In symptomatic subjects, four (11.4 %) had tibiofemoral OA and five (14.3 %) had patellofemoral OA (Table 3).

### 3.3 Background and clinical characteristics of symptomatic and asymptomatic subjects with and without MRI findings

Next, we evaluated background and clinical data in asymptomatic and symptomatic subjects with and without MRI findings (Tables 4, S4 and S5). 61.6 % of subjects had an MRI finding [cartilage loss (grade >1), osteophyte (grade >1), BML, full-thickness cartilage loss, meniscal tear or maceration, popliteal cyst, ACL or PCL tear or repair, or joint effusion (grade <1)]. 54.4 % of asymptomatic MRI-negative subjects were female compared to 88.9 % of symptomatic MRI-negative subjects. No clear difference in WOMAC scores was observed between symptomatic subjects with and without MRI findings. A family history of knee OA was more common in MRI-positive subjects, especially in the symptomatic ones. Symptomatic MRI-negative subjects were more often smokers and had lower physical activity scores (Table 4).

**Table 4:** **Background characteristics in symptomatic and asymptomatic subjects with and without MRI findings.** MRI findings include cartilage loss (grade >1), osteophyte (grade >1), any BML, any full-thickness cartilage loss, any meniscal tear or maceration, popliteal cyst, ACL and PCL tears and repairs and joint effusion (grade <1).

MRI finding	No	No	Yes	Yes
Symptoms	Asymptomatic	Symptomatic	Asymptomatic	Symptomatic
Number of participants (n)	100 (100.0)	9 (100.0)	149 (100.0)	26 (100.0)
Males n (%)	34 (34.0)	1 (11.1)	68 (45.6)	8 (30.8)
Females n (%)	66 (66.0)	8 (88.9)	81 (54.4)	18 (69.2)
Age (years)	33.6 (0.4)	33.7 (0.6)	33.7 (0.4)	33.7 (0.4)
WOMAC pain	1.2 (1.7)	10.2 (9.5)	1.7 (2.2)	10.0 (6.1)
WOMAC stiffness	0.7 (1.0)	4.3 (3.9)	0.7 (1.1)	4.5 (4.1)
WOMAC function	2.8 (4.8)	20.8 (20.7)	2.5 (4.4)	22.5 (22.3)
WOMAC total	4.6 (6.7)	34.9 (32.4)	4.7 (6.6)	36.6 (28.8)
Prior lower limb fracture n (%)	13 (13.0)	1 (11.1)	22 (14.8)	1 (4.0)
Family history of knee OA n (%)	26 (26.0)	2 (22.2)	49 (32.9)	14 (53.8)
Anti-inflammatory medication n (%)	3 (3.0)	0 (0.0)	3 (2.0)	0 (0.0)
Strong pain medication (%)	4 (4.0)	1 (11.1)	10 (6.7)	2 (7.7)
Ever smoker n (%)	53 (53.0)	7 (77.8)	100 (67.1)	16 (61.5)
Alcohol consumption g/week	27.4 (32.3)	30.5 (34.8)	35.4 (47.4)	27.9 (27.0)
Physical activity score	15.0 (3.5)	13.0 (3.7)	14.9 (3.1)	14.9 (3.7)
BMI (kg/mˆ2)	24.6 (4.1)	25.9 (4.5)	26.2 (4.7)	26.9 (5.3)
Waist circumference (cm)	83.8 (14.1)	88.3 (10.8)	88.2 (12.1)	90.5 (14.6)
Hip circumference (cm)	97.6 (13.6)	100.3 (10.8)	100.7 (9.9)	101.8 (11.6)
fP-Glucose (mmol/L)	5.0 (0.8)	4.7 (0.3)	5.0 (0.4)	5.0 (0.4)
fP-total cholesterol (mmol/L)	4.5 (0.8)	4.6 (1.3)	4.8 (1.0)	4.6 (0.7)
fP-HDL cholesterol (mmol/L)	1.5 (0.3)	1.4 (0.6)	1.5 (0.3)	1.5 (0.4)
fP-LDL cholesterol (mmol/L)	2.6 (0.7)	2.9 (1.2)	2.8 (0.9)	2.7 (0.7)
fP-triglycerides (mmol/L)	0.9 (0.5)	1.0 (0.5)	1.0 (0.9)	1.1 (0.6)
hs-CRP (mg/l)	2.1 (4.4)	4.0 (7.5)	1.2 (1.5)	1.6 (2.4)
P-Urate (umol/L)	291.2 (67.4)	287.0 (69.1)	315.8 (70.9)	303.1 (74.0)
Systolic blood pressure (mmHg)	110.9 (12.2)	106.1 (10.7)	113.0 (12.2)	112.9 (11.0)
Diastolic blood pressure (mmHg)	73.1 (9.0)	75.1 (11.4)	74.8 (8.7)	75.8 (8.8)
Heart rate (bpm)	73.8 (13.1)	78.4 (8.3)	70.6 (11.8)	72.1 (13.2)

Symptomatic = VAS >3 in any WOMAC sub-question.

Of clinical measures, BMI was higher in MRI-positive subjects, while a smaller difference was observed according to clinical symptoms. Waist and hip circumferences were significantly smaller in asymptomatic MRI-negative subjects compared to other subject groups. MRI-positive subjects had slightly higher P-Urate levels, while symptomatic MRI-negative subjects had the highest hs-CRP levels (Table 4).

### 3.4 Knee MRI findings are more common in symptomatic subjects than asymptomatic subjects

Next, we evaluated individual knee MRI findings in asymptomatic and symptomatic subjects with and without MRI findings (Table 5, Table 6). Severe cartilage lesions in both tibiofemoral and patellofemoral joints were more common in symptomatic MRI-positive subjects compared to asymptomatic MRI-positive ones. Full-thickness cartilage loss and BMLs were rare but more common in symptomatic MRI-positive subjects compared to asymptomatic MRI-positive ones. Similarly, larger osteophytes were more common in symptomatic MRI-positive subjects compared to asymptomatic MRI-positive subjects (Table 5).

**Table 5:** Counts of the most severe in-patient MRI-detected cartilage lesions, BMLs and osteophytes in the tibiofemoral and patellofemoral joint regions in symptomatic and asymptomatic subjects with and without MRI findings. MRI findings include cartilage loss (grade >1), osteophyte (grade >1), any BML, any full-thickness cartilage loss, any meniscal tear, popliteal cyst, ACL and PCL tears and repairs and joint effusion (grade <1). Symptomatic = VAS >3 in any WOMAC sub-question.

Mri finding	Tibia medial				Tibia lateral				Femur medial				Femur lateral				Patellofemoral			
	No	Yes	Yes	No	No	Yes	Yes	No	No	Yes	Yes	No	No	Yes	Yes	No	No	Yes	Yes	No
Symptoms	**−**	**−**	**+**	**+**	**−**	**−**	**+**	**+**	**−**	**−**	**+**	**+**	**−**	**−**	**+**	**+**	**−**	**−**	**+**	**+**
**Cartilage loss**																				
0 (none)	99 (99.0)	141 (94.6)	22 (84.6)	9 (100.0)	94 (94.0)	136 (91.3)	19 (73.1)	8 (88.9)	94 (94.0)	110 (73.8)	17 (65.4)	9 (100.0)	97 (97.0)	138 (92.9)	23 (88.5)	9 (100.0)	61 (61.0)	50 (33.6)	7 (26.9)	5 (55.6)
1 (<10 %)	1 (1.0)	6 (4.0)	4 (15.4)	0 (0.0)	6 (6.0)	11 (7.4)	5 (19.2)	1 (11.1)	6 (6.0)	25 (16.8)	3 (11.5)	0 (0.0)	3 (3.0)	7 (4.7)	2 (7.7)	0 (0.0)	39 (39.0)	52 (34.9)	9 (34.6)	4 (44.4)
2 (<10–75 %)	0 (0.0)	2 (1.3)	0 (0.0)	0 (0.0)	0 (0.0)	2 (1.3)	2 (7.7)	0 (0.0)	0 (0.0)	14 (9.4)	5 (19.2)	0 (0.0)	0 (0.0)	4 (2.7)	1 (3.8)	0 (0.0)	0 (0.0)	40 (26.8)	9 (34.6)	0 (0.0)
3 (>75 %)	0 (0.0)	0 (0.0)	0 (0.0)	0 (0.0)	0 (0.0)	0 (0.0)	0 (0.0)	0 (0.0)	0 (0.0)	0 (0.0)	1 (3.8)	0 (0.0)	0 (0.0)	0 (0.0)	0 (0.0)	0 (0.0)	0 (0.0)	7 (4.7)	1 (3.8)	0 (0.0)
**Full thick. c. loss**																				
0 (none)	100 (100.0)	148 (99.3)	25 (96.2)	9 (100.0)	100 (100.0)	145 (97.3)	24 (92.3)	9 (100.0)	100 (100.0)	144 (96.6)	22 (84.6)	9 (100.0)	100 (100.0)	143 (96.0)	25 (96.2)	9 (100.0)	100 (100.0)	122 (81.9)	20 (76.9)	9 (100.0)
1 (<10 %)	0 (0.0)	0 (0.0)	1 (3.8)	0 (0.0)	0 (0.0)	4 (2.7)	2 (7.7)	0 (0.0)	0 (0.0)	2 (1.3)	2 (7.7)	0 (0.0)	0 (0.0)	4 (2.7)	0 (0.0)	0 (0.0)	0 (0.0)	23 (15.4)	3 (11.5)	0 (0.0)
2 (<10–75 %)	0 (0.0)	1 (0.7)	0 (0.0)	0 (0.0)	0 (0.0)	0 (0.0)	0 (0.0)	0 (0.0)	0 (0.0)	2 (1.3)	2 (7.7)	0 (0.0)	0 (0.0)	2 (1.3)	1 (3.8)	0 (0.0)	0 (0.0)	4 (2.7)	2 (7.7)	0 (0.0)
3 (>75 %)	0 (0.0)	0 (0.0)	0 (0.0)	0 (0.0)	0 (0.0)	0 (0.0)	0 (0.0)	0 (0.0)	0 (0.0)	1 (0.7)	0 (0.0)	0 (0.0)	0 (0.0)	0 (0.0)	0 (0.0)	0 (0.0)	0 (0.0)	0 (0.0)	1 (3.8)	0 (0.0)
**Size of BML N(%)**																				
0 (none)	100 (100.0)	148 (99.3)	25 (96.2)	9 (100.0)	100 (100.0)	145 (97.3)	24 (92.3)	9 (100.0)	100 (100.0)	146 (98.0)	22 (84.6)	9 (100.0)	100 (100.0)	145 (97.3)	25 (96.2)	9 (100.0)	100 (100.0)	129 (86.6)	21 (80.8)	9 (100.0)
1 (<33 %)	0 (0.0)	0 (0.0)	1 (3.8)	0 (0.0)	0 (0.0)	4 (2.7)	2 (7.7)	0 (0.0)	0 (0.0)	3 (2.0)	2 (7.7)	0 (0.0)	0 (0.0)	3 (2.0)	1 (3.8)	0 (0.0)	0 (0.0)	17 (11.4)	3 (11.5)	0 (0.0)
2 (33–66 %)	0 (0.0)	1 (0.7)	0 (0.0)	0 (0.0)	0 (0.0)	0 (0.0)	0 (0.0)	0 (0.0)	0 (0.0)	0 (0.0)	1 (3.8)	0 (0.0)	0 (0.0)	1 (0.7)	0 (0.0)	0 (0.0)	0 (0.0)	2 (1.3)	2 (7.7)	0 (0.0)
3 (>66 %)	0 (0.0)	0 (0.0)	0 (0.0)	0 (0.0)	0 (0.0)	0 (0.0)	0 (0.0)	0 (0.0)	0 (0.0)	0 (0.0)	1 (3.8)	0 (0.0)	0 (0.0)	0 (0.0)	0 (0.0)	0 (0.0)	0 (0.0)	1 (0.7)	0 (0.0)	0 (0.0)
**% that is BML**																				
0 (none)	100 (100.0)	148 (99.3)	25 (96.2)	9 (100.0)	100 (100.0)	145 (97.3)	24 (92.3)	9 (100.0)	100 (100.0)	146 (98.0)	22 (84.6)	9 (100.0)	100 (100.0)	145 (97.3)	25 (96.2)	9 (100.0)	100 (100.0)	132 (88.6)	21 (80.8)	9 (100.0)
1 (<33 %)	0 (0.0)	0 (0.0)	0 (0.0)	0 (0.0)	0 (0.0)	0 (0.0)	0 (0.0)	0 (0.0)	0 (0.0)	0 (0.0)	0 (0.0)	0 (0.0)	0 (0.0)	0 (0.0)	0 (0.0)	0 (0.0)	0 (0.0)	0 (0.0)	0 (0.0)	0 (0.0)
2 (33–66 %)	0 (0.0)	0 (0.0)	0 (0.0)	0 (0.0)	0 (0.0)	0 (0.0)	0 (0.0)	0 (0.0)	0 (0.0)	0 (0.0)	1 (3.8)	0 (0.0)	0 (0.0)	0 (0.0)	0 (0.0)	0 (0.0)	0 (0.0)	1 (0.7)	0 (0.0)	0 (0.0)
3 (>66 %)	0 (0.0)	1 (0.7)	1 (3.8)	0 (0.0)	0 (0.0)	4 (2.7)	2 (7.7)	0 (0.0)	0 (0.0)	3 (2.0)	3 (11.5)	0 (0.0)	0 (0.0)	4 (2.7)	1 (3.8)	0 (0.0)	0 (0.0)	16 (10.7)	5 (19.2)	0 (0.0)
**Osteophyte**																				
0 (none)	100 (100.0)	135 (90.6)	21 (80.8)	9 (100.0)	97 (97.0)	136 (91.3)	20 (76.9)	9 (100.0)	100 (100.0)	138 (92.6)	19 (73.1)	9 (100.0)	94 (94.0)	123 (82.6)	20 (76.9)	9 (100.0)	63 (63.0)	61 (40.9)	8 (30.8)	6 (66.7)
1 (small)	0 (0.0)	12 (8.1)	2 (7.7)	0 (0.0)	3 (3.0)	12 (8.1)	2 (7.7)	0 (0.0)	0 (0.0)	9 (6.0)	4 (15.4)	0 (0.0)	6 (6.0)	23 (15.4)	3 (11.5)	0 (0.0)	37 (37.0)	75 (50.3)	12 (46.2)	3 (33.3)
2 (medium)	0 (0.0)	1 (0.7)	2 (7.7)	0 (0.0)	0 (0.0)	1 (0.7)	3 (11.5)	0 (0.0)	0 (0.0)	1 (0.7)	2 (7.7)	0 (0.0)	0 (0.0)	2 (1.3)	2 (7.7)	0 (0.0)	0 (0.0)	12 (8.1)	5 (19.2)	0 (0.0)
3 (large)	0 (0.0)	1 (0.7)	1 (3.8)	0 (0.0)	0 (0.0)	0 (0.0)	1 (3.8)	0 (0.0)	0 (0.0)	1 (0.7)	1 (3.8)	0 (0.0)	0 (0.0)	1 (0.7)	1 (3.8)	0 (0.0)	0 (0.0)	1 (0.7)	1 (3.8)	0 (0.0)

**Table 6:** **Meniscal morphology, other parameters of interest and prevalence and severity of knee joint effusion in the study population according to classes based on WOMAC scores and MRI findings.** MRI findings include cartilage loss (grade >1), osteophyte (grade >1), any BML, any full-thickness cartilage loss, any meniscal tear, popliteal cyst, ACL and PCL tears and repairs and joint effusion (grade <1). Symptomatic = VAS >3 in any WOMAC sub-question.

Medial meniscal morphology	Anterior				Body				Posterior							
MRI finding	**No**	**Yes**	**Yes**	**No**	**No**	**Yes**	**Yes**	**No**	**No**	**Yes**	**Yes**	**No**				
Symptoms	**−**	**−**	**+**	**+**	**−**	**−**	**+**	**+**	**−**	**−**	**+**	**+**				
Normal	100 (100.0)	144 (96.6)	24 (92.3)	9 (100.0)	93 (93.0)	125 (83.9)	19 (73.1)	9 (100.0)	93 (93.0)	127 (85.2)	18 (69.2)	9 (100.0)				
Intrameniscal signal	0 (0.0)	2 (1.3)	1 (3.8)	0 (0.0)	7 (7.0)	10 (6.7)	4 (15.4)	0 (0.0)	7 (7.0)	10 (6.7)	5 (19.2)	0 (0.0)				
Vertical tear	0 (0.0)	0 (0.0)	0 (0.0)	0 (0.0)	0 (0.0)	0 (0.0)	0 (0.0)	0 (0.0)	0 (0.0)	0 (0.0)	0 (0.0)	0 (0.0)				
Horizontal tear	0 (0.0)	1 (0.7)	0 (0.0)	0 (0.0)	0 (0.0)	10 (6.7)	1 (3.8)	0 (0.0)	0 (0.0)	10 (6.7)	1 (3.8)	0 (0.0)				
Radial tear	0 (0.0)	0 (0.0)	0 (0.0)	0 (0.0)	0 (0.0)	0 (0.0)	0 (0.0)	0 (0.0)	0 (0.0)	0 (0.0)	0 (0.0)	0 (0.0)				
Complex tear	0 (0.0)	2 (1.3)	0 (0.0)	0 (0.0)	0 (0.0)	3 (2.0)	1 (3.8)	0 (0.0)	0 (0.0)	1 (0.7)	1 (3.8)	0 (0.0)				
Partial maceration	0 (0.0)	0 (0.0)	1 (3.8)	0 (0.0)	0 (0.0)	1 (0.7)	1 (3.8)	0 (0.0)	0 (0.0)	1 (0.7)	1 (3.8)	0 (0.0)				
Total maceration	0 (0.0)	0 (0.0)	0 (0.0)	0 (0.0)	0 (0.0)	0 (0.0)	0 (0.0)	0 (0.0)	0 (0.0)	0 (0.0)	0 (0.0)	0 (0.0)				

Lateral meniscal morphology	**Anterior**				**Body**				**Posterior**							

MRI finding	**No**	**Yes**	**Yes**	**No**	**No**	**Yes**	**Yes**	**No**	**No**	**Yes**	**Yes**	**No**				

Symptoms	**−**	**−**	**+**	**+**	**−**	**−**	**+**	**+**	**−**	**−**	**+**	**+**				

Normal	99 (99.0)	146 (98.0)	26 (100.0)	9 (100.0)	100 (100.0)	147 (98.7)	25 (96.2)	9 (100.0)	98 (98.0)	147 (98.7)	25 (96.2)	9 (100.0)				
Intrameniscal signal	1 (1.0)	0 (0.0)	0 (0.0)	0 (0.0)	0 (0.0)	0 (0.0)	0 (0.0)	0 (0.0)	2 (2.0)	1 (0.7)	0 (0.0)	0 (0.0)				
Vertical tear	0 (0.0)	0 (0.0)	0 (0.0)	0 (0.0)	0 (0.0)	0 (0.0)	0 (0.0)	0 (0.0)	0 (0.0)	0 (0.0)	0 (0.0)	0 (0.0)				
Horizontal tear	0 (0.0)	2 (1.3)	0 (0.0)	0 (0.0)	0 (0.0)	1 (0.7)	0 (0.0)	0 (0.0)	0 (0.0)	1 (0.7)	1 (3.8)	0 (0.0)				
Radial tear	0 (0.0)	0 (0.0)	0 (0.0)	0 (0.0)	0 (0.0)	0 (0.0)	0 (0.0)	0 (0.0)	0 (0.0)	0 (0.0)	0 (0.0)	0 (0.0)				
Complex tear	0 (0.0)	1 (0.7)	0 (0.0)	0 (0.0)	0 (0.0)	1 (0.7)	1 (3.8)	0 (0.0)	0 (0.0)	0 (0.0)	0 (0.0)	0 (0.0)				
Partial maceration	0 (0.0)	0 (0.0)	0 (0.0)	0 (0.0)	0 (0.0)	0 (0.0)	0 (0.0)	0 (0.0)	0 (0.0)	0 (0.0)	0 (0.0)	0 (0.0)				
Total maceration	0 (0.0)	0 (0.0)	0 (0.0)	0 (0.0)	0 (0.0)	0 (0.0)	0 (0.0)	0 (0.0)	0 (0.0)	0 (0.0)	0 (0.0)	0 (0.0)				

**Other parameters of interest**	**Present**	**Present**	**Present**	**Present**												

MRI finding	**No**	**Yes**	**Yes**	**No**												

Symptoms	**−**	**−**	**+**	**+**												

ACL tear	0 (0.0)	2 (1.3)	0 (0.0)	0 (0.0)												
ACL repair	0 (0.0)	0 (0.0)	2 (7.7)	0 (0.0)												
PCL tear	0 (0.0)	2 (1.3)	1 (3.8)	0 (0.0)												
PCL repair	0 (0.0)	0 (0.0)	0 (0.0)	0 (0.0)												
Patellar tendon signal	3 (3.0)	4 (2.7)	5 (19.2)	0 (0.0)												
Any ganglion cyst	14 (14.0)	42 (28.2)	6 (23.0)	1 (11.1)												
Pes anserine bursitis	0 (0.0)	2 (1.3)	0 (0.0)	0 (0.0)												
Infrapatellar bursa signal	11 (11.0)	30 (20.1)	8 (30.8)	0 (0.0)												
Prepatellar bursa signal	32 (32.0)	52 (34.9)	9 (34.6)	2 (22.2)												
Popliteal cyst	0 (0.0)	91 (61.1)	19 (73.1)	0 (0.0)												

	**None**				**Small**				**Medium**				**Large**			

MRI finding	**No**	**Yes**	**Yes**	**No**	**No**	**Yes**	**Yes**	**No**	**No**	**Yes**	**Yes**	**No**	**No**	**Yes**	**Yes**	**No**

Symptoms	**−**	**−**	**+**	**+**	**−**	**−**	**+**	**+**	**−**	**−**	**+**	**+**	**−**	**−**	**+**	**+**

Joint effusion	73 (73.0)	75 (50.3)	8 (30.8)	6 (66.7)	27 (27.0)	56 (37.6)	13 (50.0)	2 (22.2)	0 (0.0)	17 (11.4)	3 (11.5)	1 (11.1)	0 (0.0)	1 (0.7)	2 (7.7)	0 (0.0)

Meniscal findings were more common in symptomatic MRI-positive subjects compared to asymptomatic MRI-positive ones, mostly accounted for by intrameniscal signal. Medial horizontal tears were more common in asymptomatic MRI-positive subjects than symptomatic MRI-positive subjects. Both ACL tears were asymptomatic while both repairs were symptomatic. Increased T2-weighted signal was more common in the patellar tendon of symptomatic MRI-positive subjects (19.2 %) compared to asymptomatic MRI-positive ones (2.7 %). Popliteal cysts were slightly more common (73.1 %) in symptomatic MRI-positive subjects than asymptomatic MRI-positive subjects (61.1 %). Symptomatic MRI-positive subjects had greater joint effusion than asymptomatic MRI-negative subjects (Table 6).

### 3.5 Associations between knee MRI findings and knee symptoms

Finally, we evaluated associations with individual knee MRI findings and clinical symptoms of the knee in unadjusted and adjusted logistic regression models (Table 5). Based on descriptive data and associations observed between the clinical and background parameters and symptoms, the regression models were adjusted for sex, family history of OA, BMI and the presence of other MRI findings (*i.e*. any full-thickness cartilage loss, BML or osteophyte, without using the given structural pathology twice in the model).

In unadjusted models, all medial femoral MRI findings were associated with clinical symptoms. Both tibiofemoral full-thickness cartilage losses and BMLs had relatively wide confidence intervals. Most osteophytes were also associated with clinical symptoms. Of other MRI findings, patellar tendon signal, popliteal cysts and joint effusion were associated with clinical symptoms (Table 7).

**Table 7:** **Associations between knee MRI findings and knee symptoms.** The ORs represent the risk of a single grade increase in the given MRI finding for having symptoms (VAS >3 in any sub-question of WOMAC, n = 35). The unadjusted model includes only the given individual MRI finding as an explanatory variable. The adjusted models were adjusted for sex, family history of OA, BMI and the presence of other MRI findings (i.e. any full-thickness cartilage loss, any BMLs, any osteophyte, without using the given structural pathology twice in the model).

Unadjusted model	Tibia medial		Tibia lateral		Femur medial		Femur lateral		Tibiofemoral		Patellofemoral		Any	
	OR (95 % CI)	*P*	OR (95 % CI)	*P*	OR (95 % CI)	*P*	OR (95 % CI)	*P*	OR (95 % CI)	*P*	OR (95 % CI)	*P*	OR (95 % CI)	*P*
Cartilage loss	2.2 (0.8–6.2)	0.15	**2.9 (1.4–6.2)**	0.004	**1.7 (1.0–2.7)**	0.044	1.5 (0.7–3.4)	0.28	**1.6 (1.0–2.6)**	0.035	1.3 (0.9–1.9)	0.18	1.3 (0.9–2.0)	0.23
Full thick. cartilage loss	2.3 (0.3–13.7)	0.43	3.7 (0.7–21.1)	0.14	**2.3 (1.0–5.0)**	0.037	1.4 (0.4–4.9)	0.56	**2.0 (1.0–4.0)**	0.037	**1.8 (1.0–3.6)**	**0.049**	**1.8 (1.0–3.0)**	**0.036**
Size of BML	2.1 (0.3–13.7)	0.43	3.7 (0.7–21.1)	0.14	**7.0 (1.7–30.0)**	0.008	1.3 (0.2–8.3)	0.78	**2.7 (1.2–5.8)**	0.012	1.7 (0.8–3.5)	0.15	**1.9 (1.1–3.3)**	**0.032**
% of lesion that is BML	**1.9 (0.8–4.9)**	0.16	1.5 (0.9–2.8)	0.14	**2.2 (1.3–3.7)**	0.004	1.22 (0.6–2.6)	0.60	**1.5 (1.0–2.2)**	0.038	1.3 (0.9–1.9)	0.12	1.3 (0.9–1.8)	0.054
Osteophyte	**2.3 (1.2–4.4)**	0.016	**3.1 (1.5–6.2)**	0.002	**2.9 (1.5–5.9)**	0.002	1.7 (0.9–3.1)	0.097	1.7 (0.9–3.0)	0.060	**1.7 (1.0–2.9)**	**0.038**	1.7 (0.9–2.8)	0.053
**Adjusted model**														
Cartilage loss	0.88 (0.24–3.29)	0.85	2.35 (0.99–5.58)	0.053	1.13 (0.60–2.13)	0.75	1.05 (0.39–2.82)	0.92	1.20 (0.67–2.16)	0.54	1.03 (0.64–1.67)	0.91	0.93 (0.55–1.56)	0.77
Full thick. cartilage loss	0.57 (0.07–4.61)	0.57	2.88 (0.45–18.4)	0.26	1.35 (0.50–3.63)	0.56	0.71 (0.15–3.46)	0.68	1.35 (0.55–3.30)	0.51	1.23 (0.46–3.30)	0.67	1.10 (0.37–3.22)	0.87
Size of BML	0.61 (0.08–4.76)	0.64	2.87 (0.43–19.2)	0.28	**7.17 (1.01–50.6)**	0.048	1.00 (0.13–7.53)	0.99	1.81 (0.58–5.58)	0.31	1.24 (0.41–3.71)	0.71	1.51 (0.44–5.13)	0.51
% of lesion that is BML	1.22 (0.37–4.00)	0.75	1.38 (0.73–2.64)	0.32	1.99 (0.90–4.39)	0.089	1.03 (0.47–2.27)	0.95	1.18 (0.69–2.02)	0.54	0.99 (0.60–1.63)	0.96	0.93 (0.49–1.73)	0.81
Osteophyte	1.72 (0.76–3.89)	0.19	**2.49 (1.11–5.59)**	0.027	**2.71 (1.17–6.31)**	0.020	1.19 (0.57–2.49)	0.65	1.32 (0.67–2.59)	0.42	1.59 (0.88–2.87)	0.13	1.50 (0.83–2.69)	0.18
**Unadjusted model**														
Med menisci tear/maceration	1.46 (0.40–5.33)	0.57												
**Patellar tendon signal**	**5.76 (1.72–19.3)**	**0.005**												
Any ganglion cyst	0.86 (0.36–2.07)	0.74												
Infrapatellar bursa signal	1.50 (0.64–3.54)	0.35												
Prepatellar bursa signal	0.90 (0.42–1.93)	0.79												
**Popliteal cyst**	**2.07 (1.01–4.21)**	**0.047**												
**Joint effusion**	**1.92 (1.21–3.06)**	**0.006**												
**Adjusted model**														
Med menisci tear/maceration	0.91 (0.21–3.88)	0.90												
**Patellar tendon signal**	**5.82 (1.58–21.4)**	**0.008**												
Any ganglion cyst	0.64 (0.24–1.69)	0.37												
Infrapatellar bursa signal	1.03 (0.39–2.72)	0.95												
Prepatellar bursa signal	0.69 (0.31–1.58)	0.38												
Popliteal cyst	1.97 (0.94–4.13)	0.072												
**Joint effusion**	**2.07 (1.20–3.56)**	**0.009**												

In the adjusted models, cartilage lesions were not significantly associated with clinical symptoms, although the confidence intervals of full-thickness cartilage losses were very wide. Medial femoral BMLs and osteophytes as well as lateral tibial osteophytes were associated with clinical symptoms. Of other MRI findings, patellar tendon signal and joint effusion were associated with clinical symptoms (Table 7).

## 4 Discussion

This study evaluated the associations of clinical parameters and individual structural MRI findings with knee symptoms in the population-based birth cohort (NFBC1986) without obvious knee OA risk factors, at 33 years of age. Of the clinical parameters, female sex, family history and to some extent adverse body composition and lipid profile were associated with advanced clinical symptoms of the knee. Most MRI findings were more common and severe in symptomatic subjects compared to the asymptomatic. The “usual suspects”, namely higher-grade and full-thickness cartilage lesions and BMLs, were overall rare in this young study population. In the adjusted models, medial and lateral femoral osteophytes and medial femoral BMLs were the MRI findings associated with symptoms, while cartilage lesions were not. The most reported symptom from the WOMAC catalogue was pain when using stairs, being one of the most common symptoms in patellofemoral pain [34].

Traditionally, articular cartilage has been viewed as the central feature of knee OA, although imaging pathology can be found in any adjacent structures. Cartilage tissue itself is aneural, and inflammatory or structural damage to the surrounding innervated tissues, such as subchondral bone and synovium and other soft tissues, are thought to be the structural causes of pain [35]. Whereas structural pathology can be demonstrated with imaging, peripheral and central sensitization can amplify the subjective experience of pain, ultimately forming a multifactorial biopsychosocial concept [35]. Having acknowledged that musculoskeletal pain is subjective and its research complex, clear associations with individual structural imaging findings have been previously reported [[12], [13], [14], [15], [16], [17], [18], [19], [20], [21]].

In our recent study on this population, higher BMI was the health factor associated with most individual knee MRI parameters [21]. Symptomatic subjects had on average higher BMI (+1.2 kg/m^2^) and greater waist circumference (+3.5 cm) with more pronounced differences observed in female participants (+1.3 kg/m^2^ and +5.3 cm, respectively). Although no clear association was detected, descriptive data suggests that higher BMI is associated with symptoms, in subjects both with and without (1.3 kg/m^2^) MRI findings (0.7 kg/m^2^). Perhaps more importantly, the data suggests that not only is BMI associated with clinical symptoms but also with overall imaging findings regardless of symptoms, highlighting the importance of weight management for both prevention and management of symptomatic OA already in young adults. This interpretation is also supported by other associations with knee symptoms, most notably those observed for higher triglyceride levels in females, and lower HDL cholesterol in males, as dyslipidemia is well-established to be associated with obesity [36].

Another observation is that 74.3 % of symptomatic subjects were females compared to 59.0 % of asymptomatic subjects. A possible explanation is greater pain sensitivity and increased risk for clinical pain among females [37]. Meanwhile, WOMAC scores were higher in symptomatic males compared to females. Furthermore, as a staggering 88.9 % of symptomatic subjects without obvious MRI findings were female, and oppositely the proportion of males was the highest in asymptomatic subjects with MRI findings (45.6 %), our data suggests that a definite difference in the interpretation of knee symptoms exists between sexes.

Having a family history of OA was more common in symptomatic females (50.0 %) compared to 27.9 % in asymptomatic females, a difference unobserved in males. Moreover, having a family history of OA was more common especially in symptomatic females with MRI findings (61.1 %) compared to symptomatic females without MRI findings (25.0 %). As such differences were observed in asymptomatic females on a much smaller scale (22.7 % in those with MRI findings compared to 32.1 % in those without), the data could suggest having a family history of OA is especially associated with clinical OA symptoms. As to why the association was only observed in females is left for speculation. Literature suggests that the heritability of OA is greater in females suggesting a genetic mechanism [38]. This was not observed here as having a family history of OA was as common in males as females [21]. As family history was self-reported, rather than actual medical history, another possible explanation is that symptomatic females rather than males, might be more aware of the medical history of their relatives, as they are more aware of their own medical conditions [39]. However, being self-reported data, it is also possible that family history is an explanation of clinical symptoms, rather than an actual cause. Overall, current literature provides possible explanations for these results but further studies with more specific study questions are required to validate and elaborate these findings.

12.2 % of all participants were symptomatic, the most common symptoms being pain using stairs (57 % of all symptomatic participants) as well as day- and nighttime knee joint stiffness 26 % and 29 %, respectively [21]. Varying estimations of reported knee pain in adolescents and young adults exist, with results ranging from 6.4 % up to 31.8 % between study populations [[40], [41], [42], [43]]. Pain using stairs is a typically reported symptom in patellofemoral pain [34] and is, in addition to joint stiffness, also associated with isolated patellofemoral OA in the elderly [44]. The overall symptomatology of the studied NFBC1986 subpopulation alings with the fact that most MRI findings are located in the patellofemoral compartment [21]. Follow-up of this NFBC1986 subpopulation will certainly be informative on the proportion of progressive cartilage lesions (only 3.5 % of participants had MRI-defined patellofemoral OA [21,24]) and the potential development of new knee joint symptoms [16].

Although the number of advanced findings in this study population is low, the association of medial femoral findings (BMLs and osteophytes) is consistent with the prior knowledge of the medial compartment being the most load bearing [45] and the reported associations between pain and BMLs [11,15,19]. Although cartilage lesions weren't associated with symptoms, medial tibial cartilage lesions at baseline as well as progressive patellar cartilage lesions have been previously associated with new knee pain symptom at 48-month time point [16]. Furthermore, knee joint effusion has been repeatedly linked with symptoms, although its potentially either reactive or synovitic origin complicates its interpretation [[13], [14], [15], [16]]. MRI signal abnormalities of the patellar tendon are usually related to patellar tendinopathy of uncertain clinical relevance, with proximal T2-weighted signal being a common imaging finding [46]. In our original grading [21], we regarded the patellar tendon to be abnormal only if abnormality was seen in both T1-and T2-weighed sequences, with or without tendon thickening and surrounding soft tissue edema. Although graded as either absent or present [30], our grading therefore considers only “moderate” or “severe” tendinopathy as present abnormality.

There are also limitations in our study, some of which have been discussed earlier [21]. First, whereas anthropometric measurements were included in the study protocol, a routine clinical examination of the knee joint was not [34]. We also do not have data on pain management at the time of the WOMAC questionnaire was conducted. However, the total number of pain medication users was low at the time of other clinical examinations. Additionally, the WOMAC questionnaire does not include questions regarding, for example, the multifocality or the duration of symptoms. A multi-step approach [25] or diary recording methods [47] could improve the identification of truly symptomatic knee joints. Sex differences in pain sensitization and coping efficacy have also been reported [48]. A clear limitation is the lack of advanced MRI findings in this study population. Full-length standing radiographs for assessing axial alignment were also unavailable for this study. Statistically speaking, assessing clinical symptoms based on a single cut-off value (such as VAS >3) is not necessarily optimal, as it decreases variance and could lead to misinterpretation of the results. However, we believe that this approach gives the most reliable estimate of the count of symptomatic subjects, as small changes on the VAS scale are arbitrary (especially at lower scores), possibly leading to false positive findings in the data if addressing symptoms on a continuous scale. The educational level and ethnicity were not assessed in this study [5]. Family history is self-reported, which might introduce recall bias. As of MRI data, a distinction between synovitis and joint effusion was not attempted as only non-enhanced MRI sequences were available [14]. Furthermore, Hoffa-synovitis was not evaluated due to its non-specificity and reportedly low intra-rater kappa scores, although its potential as an MRI biomarker is recognized [30,31]. Finally, as MOAKS does not evaluate the static MRI features of patellofemoral alignment such as features of trochlear dysplasia, patellar translation, patellar height and rotational asymmetry [49], patellofemoral instability [50] as a potential confounder was not evaluated. However, we are planning on evaluating these MRI features in the future.

In conclusion, in this random subsample of a birth cohort at 33 years of age, several MRI findings, most notably medial femoral BMLs, osteophytes, patellar tendinopathy and joint effusion were associated with knee symptoms. Of other factors, sex, family history and clinical factors such as body composition are associated with knee joint symptomatology from early adulthood.

## Author contributions

A.K.: Conceptualization, data curation, writing - original draft, writing – review & editing.

J.T.: Conceptualization, formal analysis, visualization, writing – original draft, writing – review & editing.

M.T. Ni: Funding acquisition, resources, writing – review & editing.

S·S.: Funding acquisition, resources, writing – review & editing.

M.T.Ne: Conceptualization, project administration, resources, supervision, writing – review & editing.

The corresponding author (A.K.) takes responsibility for the overall integrity of the research article.

## Funding source

This work was supported by the Research Council of Finland (Flagship of Advanced Mathematics for Sensing Imaging and Modelling grant #359186 and grant #354692). NFBC1986 33-35y follow-up study received financial support from University of Oulu (Strategic funding from donations) and Oulu University Hospital (K65760).

## Conflict of interest

The authors declare no conflict of interest.

## Figures and Tables

**Supplementary figures 1:**