# Supplementary material for: Associations of clinical measures and structural knee magnetic resonance imaging findings with knee symptoms in a birth cohort of 33-year-old adults
Source: Osteoarthr Cartil Open. 2025 Dec 13;8(1):100730. doi: 10.1016/j.ocarto.2025.100730 (PMC12800690; doi:10.1016/j.ocarto.2025.100730)
Supplement: Multimedia component 1 [file mmc1.docx]

**Associations of clinical measures and structural knee MRI findings with knee symptoms in a birth cohort of 33-year-old adults**

Antti Kemppainen^a,b*¤^, Joona Tapio^c,d¤^, Miika Nieminen^a,b,e^, Simo Saarakkala^a,b,d,e^, Mika T. Nevalainen^a,b,e^

^a^Research Unit of Health Sciences and Technology

Faculty of Medicine, University of Oulu

P.O. Box 5000, FI-90014 Oulu, Finland

^b^Department of Diagnostic Radiology

Oulu University Hospital

P.O. Box 50, FI-90029 Oulu, Finland

^c^Faculty of Biochemistry and Molecular Medicine, University of Oulu

P.O. Box 5400, FIN-90014 Oulu, Finland

^d^Biocenter Oulu, University of Oulu

P.O. Box 5400, FIN-90014 Oulu, Finland

^e^Medical Research Center Oulu

University of Oulu and Oulu University Hospital

Oulu, Finland

*Corresponding author

Antti Kemppainen

E-mail address: [antti.kemppainen@pohde.fi](mailto:antti.kemppainen@pohde.fi)

¤ Equal contribution

**Supplementary data**

**Figure S1. Flow chart representing the study population and analyses done.** NFBC1986: Northern Finland Birth Cohort, MRI; Magnetic Resonance Imaging, OA; osteoarthritis, BML; Bone Marrow Lesion, ACL; Anterior Cruciate Ligament, PCL; Posterior Cruciate Ligament, BMI; Body Mass Index, fP-; fasted plasma, HDL; High-Density Lipoprotein, LDL; Low-Density Lipoprotein**,** hs-CRP: high-sensitivity C-Reactive Protein, Alat; alanine aminotransferase, WOMAC; Western Ontario and McMaster Universities Osteoarthritis Index


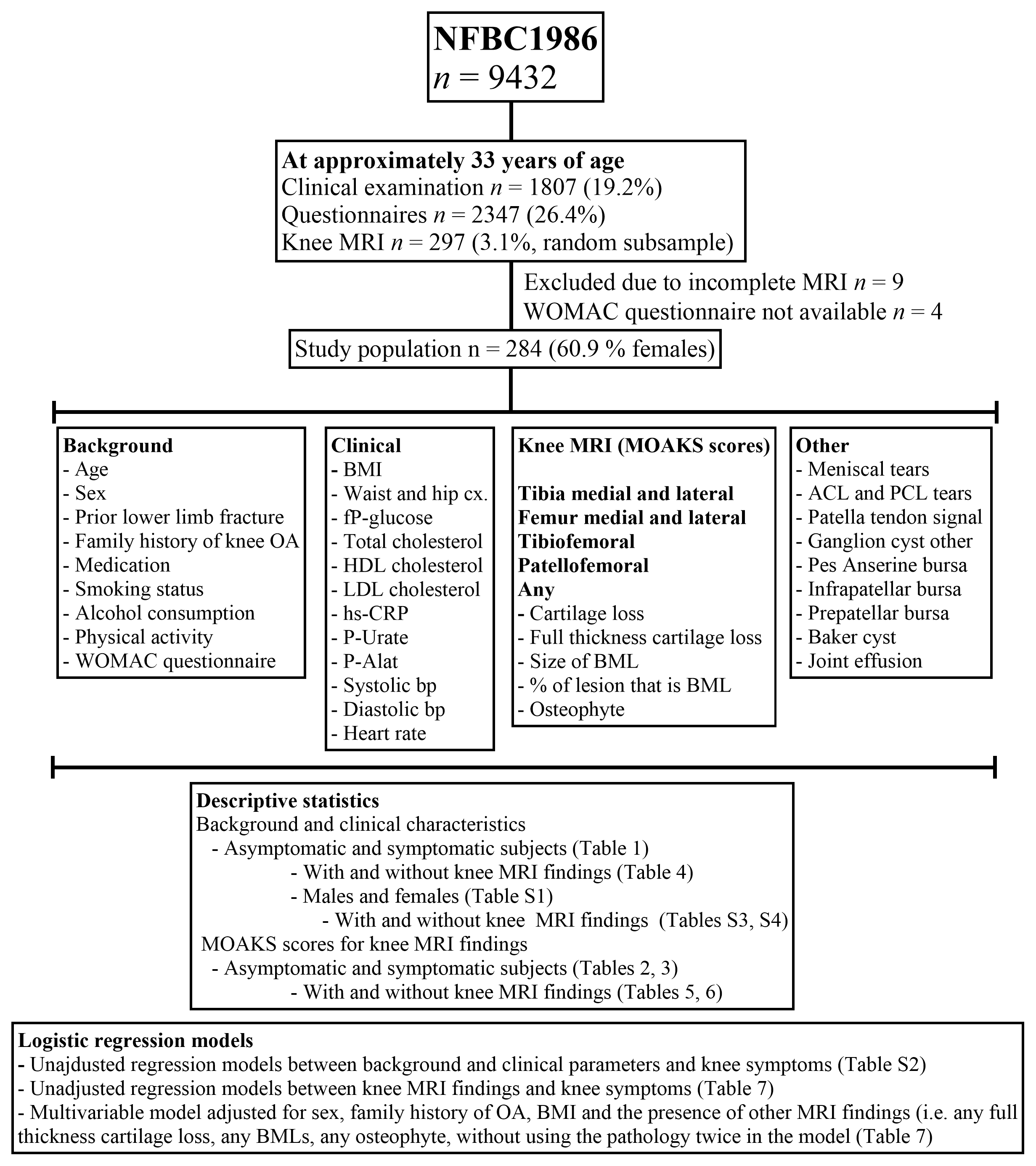


|  | **Asymptomatic males** | **Symptomatic males** | **Asymptomatic females** | **Symptomatic females** |
| --- | --- | --- | --- | --- |
| **Number of participants n (%)** | 102 (91.8) | 9 (8.2) | 147 (85.0) | 26 (15.0) |
| **Age (years)** | 33.7 (0.4) | 33.7 (0.5) | 33.7 (0.3) | 33.7 (0.4) |
| **WOMAC Pain** | 1.3 (1.8) | 11.2 (8.9) | 1.6 (2.2) | 9.8 (6.4) |
| **WOMAC Stiffness** | 0.6 (0.9) | 4.7 (5.3) | 0.7 (1.1) | 4.4 (3.6) |
| **WOMAC Function** | 2.3 (4.2) | 28.7 (33.6) | 2.8 (4.8) | 19.7 (15.8) |
| **WOMAC Total** | 4.1 (5.9) | 43.9 (44.1) | 5.0 (7.1) | 33.5 (22.8) |
| **Prior lower limb fracture n (%)** | 20 (19.6) | 0 (0.0) | 15 (10.2) | 2 (7.7) |
| **Family history of knee OA n (%)** | 34 (33.3) | 3 (33.3) | 41 (27.9) | 13 (50.0) |
| **Anti-inflammatory medication n (%)** | 3 (2.9) | 0 (0.0) | 3 (2.0) | 0 (0.0) |
| **Ever smoker n (%)** | 69 (67.6) | 6 (66.7) | 84 (57.1) | 17 (65.4) |
| **Alcohol consumption g/week** | 48.5 (53.5) | 48.4 (32.8) | 21.0 (27.2) | 22.3 (24.9) |
| **Physical activity score** | 14.9 (3.6) | 14.0 (4.4) | 14.9 (3.0) | 14.6 (3.7) |
| **BMI (kg/m^2)** | 25.6 (3.7) | 26.3 (3.1) | 25.5 (5.0) | 26.8 (5.6) |
| **Waist circumference (cm)** | 91.5 (10.0) | 94.9 (9.9) | 82.9 (13.9) | 88.2 (14.4) |
| **Hip circumference (cm)** | 100.1 (6.5) | 100.1 (5.7) | 98.9 (14.1) | 101.9 (12.7) |
| **fP-Glucose (mmol/L)** | 5.2 (0.4) | 5.2 (0.4) | 4.9 (0.7) | 4.8 (0.4) |
| **fP-Total cholesterol (mmol/L)** | 4.9 (0.9) | 4.8 (0.6) | 4.5 (0.8) | 4.6 (1.0) |
| **fP-HDL cholesterol (mmol/L)** | 1.4 (0.3) | 1.2 (0.2) | 1.6 (0.3) | 1.6 (0.4) |
| **fP-LDL cholesterol (mmol/L)** | 3.0 (0.8) | 3.2 (0.7) | 2.5 (0.7) | 2.6 (0.9) |
| **fP-Triglycerides (mmol/L)** | 1.1 (1.0) | 1.2 (0.5) | 0.8 (0.4) | 1.0 (0.6) |
| **hs-CRP (mg/l)** | 0.9 (1.4) | 0.8 (0.7) | 2.0 (3.7) | 2.8 (4.9) |
| **P-Urate (umol/L)** | 352.9 (59.6) | 353.4 (43.9) | 273.3 (57.9) | 278.3 (70.3) |
| **Systolic blood pressure (mmHg)** | 120.0 (10.4) | 120.3 (11.6) | 106.7 (10.4) | 108.0 (9.5) |
| **Diastolic blood pressure (mmHg)** | 76.3 (8.5) | 78.9 (7.2) | 72.6 (8.8) | 74.5 (9.9) |
| **Heart rate (bpm)** | 69.9 (14.2) | 71.4 (7.5) | 73.3 (10.8) | 74.5 (13.7) |

**Table S1. Background characteristics of symptomatic and asymptomatic males and females of the study population.** Asymptomatic = VAS < 3 in all WOMAC sub-questions, symptomatic = VAS > 3 in any WOMAC sub-question.

|  | **Unadjusted model** | | **Unadjusted model males** | | **Unadjusted model females** | |
| --- | --- | --- | --- | --- | --- | --- |
|  | **OR (95% CI)** | ***P*** | **OR (95% CI)** | ***P*** | **OR (95% CI)** | ***P*** |
| **Sex (female)** | 2.01 (0.90 - 4.46) | 0.088 | N/A | N/A | N/A | N/A |
| **Age (years)** | 0.85 (0.32 - 2.26) | 0.745 | 0.83 (0.14 - 5.02) | 0.837 | 0.89 (0.27 - 2.91) | 0.851 |
| **Prior lower limb fracture n (%)** | 0.38 (0.09 - 1.67) | 0.200 | N/A | N/A | 0.77 (0.16 - 3.57) | 0.734 |
| **Family history of knee OA n (%)** | 1.95 (0.95 - 4.00) | 0.068 | 1.00 (0.24 - 4.25) | 1.000 | 2.59 (1.11 - 6.04) | 0.028 |
| **Ever smoker n (%)** | 1.20 (0.57 - 2.53) | 0.627 | 0.96 (0.23 - 4.06) | 0.952 | 1.42 (0.59 - 3.39) | 0.433 |
| **Alcohol consumption g/week** | 1.00 (0.99 - 1.01) | 0.642 | 1.00 (0.99 - 1.01) | 0.994 | 1.00 (0.99 - 1.02) | 0.824 |
| **Physical activity score** | 0.96 (0.87 - 1.07) | 0.440 | 0.94 (0.79 - 1.12) | 0.492 | 0.97 (0.85 - 1.11) | 0.623 |
| **BMI (kg/m^2)** | 1.05 (0.98 - 1.13) | 0.173 | 1.05 (0.88 - 1.26) | 0.587 | 1.05 (0.97 - 1.13) | 0.235 |
| **Waist circumference (cm)** | 1.02 (0.99 - 1.05) | 0.142 | 1.03 (0.97 - 1.10) | 0.321 | 1.03 (0.99 - 1.06) | 0.076 |
| **Hip circumference (cm)** | 1.02 (0.98 - 1.05) | 0.332 | 1.00 (0.90 - 1.11) | 0.970 | 1.02 (0.98 - 1.05) | 0.310 |
| **fP-Glucose (mmol/L)** | 0.79 (0.36 - 1.75) | 0.566 | 1.08 (0.17 - 6.90) | 0.937 | 0.95 (0.48 - 1.89) | 0.891 |
| **fP-Total cholesterol (mmol/L)** | 0.95 (0.64 - 1.41) | 0.780 | 0.81 (0.37 - 1.75) | 0.587 | 1.14 (0.71 - 1.84) | 0.590 |
| **fP-HDL cholesterol (mmol/L)** | 0.88 (0.30 - 2.56) | 0.811 | 0.03 (0.01 - 0.74) | 0.031 | 1.28 (0.37 - 4.44) | 0.699 |
| **fP-LDL cholesterol (mmol/L)** | 1.03 (0.67 - 1.59) | 0.894 | 1.28 (0.55 - 2.97) | 0.562 | 1.13 (0.66 - 1.93) | 0.652 |
| **fP-Triglycerides (mmol/L)** | 1.19 (0.80 - 1.78) | 0.399 | 1.03 (0.54 - 1.98) | 0.921 | 2.66 (1.11 - 6.42) | 0.029 |
| **hs-CRP (mg/l)** | 1.05 (0.97 - 1.15) | 0.245 | 0.91 (0.50 - 1.66) | 0.751 | 1.04 (0.95 - 1.14) | 0.371 |
| **P-Urate (umol/L)** | 1.00 (0.99 - 1.00) | 0.514 | 1.00 (0.99 - 1.01) | 0.978 | 1.00 (0.99 - 1.01) | 0.696 |
| **Systolic blood pressure (mmHg)** | 0.99 (0.96 - 1.02) | 0.665 | 1.00 (0.94 - 1.07) | 0.922 | 1.01 (0.97 - 1.05) | 0.540 |
| **Diastolic blood pressure (mmHg)** | 1.02 (0.98 - 1.06) | 0.352 | 1.04 (0.96 - 1.12) | 0.371 | 1.02 (0.98 - 1.07) | 0.330 |
| **Heart rate (bpm)** | 1.01 (0.98 - 1.04) | 0.413 | 1.01 (0.96 - 1.06) | 0.738 | 1.01 (0.97 - 1.05) | 0.614 |

**Table S2. Odds ratios of individual background parameters in unadjusted logistic regression model for having knee symptoms.** The ORs are given for having VAS > 3 in any sub-question of WOMAC (*n* = 35). N/A; not applicable

| KL grade | Medial TF joint n (%) | Lateral TF joint n (%) | TF joint n (%) |
| --- | --- | --- | --- |
| 0 | 251 (87.2) | 264 (91.7) | 240 (83.3) |
| 1 | 31 (10.8) | 19 (6.6) | 41 (14.2) |
| 2 | 4 (1.4) | 5 (1.7) | 5 (1.7) |
| 3 | 2 (0.7) | 0 (0.0) | 2 (0.7) |
| 4 | 0 (0.0) | 0 (0.0) | 0 (0.0) |

**Table S3. Kellgren-Lawrence grades graded from radiographs of the MRI-imaged knees.** Total *n* = 288, 4 participants had missing WOMAC data. KL; Kellgren-Lawrence, TF; tibiofemoral.

| **MRI finding** | **No** | **No** | **Yes** | **Yes** |
| --- | --- | --- | --- | --- |
| **Symptoms** | **Asymptomatic** | **Symptomatic** | **Asymptomatic** | **Symptomatic** |
| **Number of participants (n)** | 34 (100.0) | 1 (100.0) | 68 (100.0) | 8 (100.0) |
| **Age (years)** | 33.6 (0.5) | 33.1 | 33.7 (0.3) | 33.7 (0.4) |
| **WOMAC Pain** | 1.0 (1.5) | 8.0 | 1.5 (2.0) | 11.6 (9.4) |
| **WOMAC Stiffness** | 0.9 (1.1) | 3.0 | 0.5 (0.7) | 4.9 (5.6) |
| **WOMAC Function** | 2.5 (4.0) | 10.0 | 2.2 (4.2) | 31.0 (35.1) |
| **WOMAC Total** | 4.2 (5.8) | 20.0 | 4.0 (6.0) | 46.9 (46.2) |
| **Prior lower limb fracture n (%)** | 7 (20.6) | 0 (0.0) | 13 (19.1) | 0 (0.0) |
| **Family history of knee OA n (%)** | 11 (32.4) | 0 (0.0) | 23 (33.8) | 3 (37.5) |
| **Anti-inflammatory medication n (%)** | 1 (2.9) | 0 (0.0) | 2 (2.9) | 0 (0.0) |
| **Ever smoker n (%)** | 19 (55.9) | 1 (100.0) | 50 (73.5) | 5 (62.5) |
| **Alcohol consumption g/week** | 40.6 (41.0) | 105.0 | 52.8 (59.0) | 40.3 (25.4) |
| **Physical activity score** | 15.0 (3.9) | 15.0 | 14.8 (3.6) | 13.9 (4.6) |
| **BMI (kg/m^2)** | 25.1 (3.2) | 25.1 | 25.8 (3.9) | 26.4 (3.3) |
| **Waist circumference (cm)** | 90.7 (8.7) | 93.5 | 91.8 (10.7) | 95.1 (10.6) |
| **Hip circumference (cm)** | 99.9 (6.1) | 102.5 | 100.2 (6.7) | 99.8 (6.0) |
| **fP-Glucose (mmol/L)** | 5.2 (0.4) | 4.9 | 5.1 (0.3) | 5.2 (0.4) |
| **fP-Total cholesterol (mmol/L)** | 4.9 (0.9) | 4.4 | 5.0 (1.0) | 4.8 (0.6) |
| **fP-HDL cholesterol (mmol/L)** | 1.5 (0.4) | 1.1 | 1.4 (0.3) | 1.2 (0.2) |
| **fP-LDL cholesterol (mmol/L)** | 3.0 (0.7) | 2.8 | 3.1 (0.8) | 3.2 (0.7) |
| **fP-Triglycerides (mmol/L)** | 1.0 (0.5) | 1.1 | 1.2 (1.2) | 1.2 (0.5) |
| **hs-CRP (mg/l)** | 0.8 (1.5) | 1.1 | 0.9 (1.4) | 0.7 (0.7) |
| **P-Urate (umol/L)** | 335.7 (54.4) | 373.0 | 361.5 (60.7) | 351.0 (46.2) |
| **Systolic blood pressure (mmHg)** | 119.8 (9.0) | 121.0 | 120.1 (11.1) | 120.3 (12.3) |
| **Diastolic blood pressure (mmHg)** | 76.1 (8.0) | 88.0 | 76.3 (8.7) | 77.8 (6.7) |
| **Heart rate (bpm)** | 73.5 (16.7) | 80.0 | 68.0 (12.5) | 70.4 (7.3) |

**Table S4. Background characteristics in symptomatic and asymptomatic male subjects with and without MRI findings.** MRI findings include cartilage loss (grade >1), osteophyte (grade >1), any BML, any full thickness cartilage loss, any meniscal tear or maceration, popliteal cyst, ACL and PCL tears and repairs and joint effusion (grade <1). Symptomatic = VAS > 3 in any WOMAC sub-question.

| **MRI finding** | **No** | **No** | **Yes** | **Yes** |
| --- | --- | --- | --- | --- |
| **Symptoms** | **Asymptomatic** | **Symptomatic** | **Asymptomatic** | **Symptomatic** |
| **Number of participants (n)** | 66 (100.0) | 8 (100.0) | 81 (100.0) | 18 (100.0) |
| **Age (years)** | 33.6 (0.3) | 33.7 (0.5) | 33.7 (0.4) | 33.6 (0.3) |
| **WOMAC Pain** | 1.3 (1.7) | 10.5 (10.1) | 1.9 (2.4) | 9.5 (4.1) |
| **WOMAC Stiffness** | 0.6 (0.9) | 4.5 (4.1) | 0.8 (1.3) | 4.3 (3.5) |
| **WOMAC Function** | 3.0 (5.2) | 22.1 (21.7) | 2.7 (4.5) | 18.7 (13.1) |
| **WOMAC Total** | 4.8 (7.2) | 36.8 (34.2) | 5.2 (7.0) | 32.1 (16.6) |
| **Prior lower limb fracture n (%)** | 6 (9.1) | 1 (12.5) | 9 (11.1) | 1 (5.6) |
| **Family history of knee OA n (%)** | 15 (22.7) | 2 (25.0) | 26 (32.1) | 11 (61.1) |
| **Anti-inflammatory medication n (%)** | 2 (3.0) | 0 (0.0) | 1 (1.2) | 0 (0.0) |
| **Ever smoker n (%)** | 34 (51.5) | 6 (75.0) | 50 (61.7) | 11 (61.1) |
| **Alcohol consumption g/week** | 20.1 (23.7) | 21.2 (22.2) | 21.8 (29.7) | 22.9 (26.7) |
| **Physical activity score** | 14.9 (3.3) | 12.8 (3.9) | 14.9 (2.7) | 15.4 (3.4) |
| **BMI (kg/m^2)** | 24.4 (4.5) | 26.0 (4.8) | 26.5 (5.3) | 27.2 (6.1) |
| **Waist circumference (cm)** | 80.2 (15.1) | 87.6 (11.4) | 85.1 (12.5) | 88.4 (15.9) |
| **Hip circumference (cm)** | 96.3 (16.1) | 100.0 (11.5) | 101.0 (12.0) | 102.7 (13.4) |
| **fP-Glucose (mmol/L)** | 4.9 (1.0) | 4.7 (0.3) | 4.8 (0.4) | 4.9 (0.4) |
| **fP-Total cholesterol (mmol/L)** | 4.3 (0.7) | 4.6 (1.4) | 4.6 (0.9) | 4.5 (0.7) |
| **fP-HDL cholesterol (mmol/L)** | 1.6 (0.3) | 1.5 (0.6) | 1.6 (0.3) | 1.7 (0.3) |
| **fP-LDL cholesterol (mmol/L)** | 2.4 (0.6) | 2.9 (1.3) | 2.6 (0.8) | 2.5 (0.6) |
| **fP-Triglycerides (mmol/L)** | 0.8 (0.4) | 1.0 (0.6) | 0.8 (0.4) | 1.0 (0.6) |
| **hs-CRP (mg/l)** | 2.8 (5.1) | 4.4 (8.0) | 1.4 (1.6) | 2.1 (2.8) |
| **P-Urate (umol/L)** | 268.3 (62.1) | 276.3 (65.4) | 277.4 (54.4) | 279.2 (74.1) |
| **Systolic blood pressure (mmHg)** | 106.3 (11.1) | 104.3 (9.7) | 107.1 (9.8) | 109.7 (9.1) |
| **Diastolic blood pressure (mmHg)** | 71.5 (9.1) | 73.5 (11.1) | 73.5 (8.5) | 74.9 (9.7) |
| **Heart rate (bpm)** | 73.9 (10.8) | 78.3 (8.8) | 72.8 (10.7) | 72.8 (15.3) |

**Table S5. Background characteristics in symptomatic and asymptomatic female subjects with and without MRI findings.** MRI findings include cartilage loss (grade >1), osteophyte (grade >1), any BML, any full thickness cartilage loss, any meniscal tear or maceration, popliteal cyst, ACL and PCL tears and repairs and joint effusion (grade <1). Symptomatic = VAS > 3 in any WOMAC sub-question.
